# Data Quality Issues With Physician-Rating Websites: Systematic Review

**DOI:** 10.2196/15916

**Published:** 2020-09-28

**Authors:** Pavankumar Mulgund, Raj Sharman, Priya Anand, Shashank Shekhar, Priya Karadi

**Affiliations:** 1 School of Management State University of New York Buffalo Buffalo, NY United States; 2 Institute of Computational and Data Sciences State University of New York Buffalo Buffalo, NY United States

**Keywords:** physician-rating websites, data quality issues, doctor ratings, reviews, data quality framework

## Abstract

**Background:**

In recent years, online physician-rating websites have become prominent and exert considerable influence on patients’ decisions. However, the quality of these decisions depends on the quality of data that these systems collect. Thus, there is a need to examine the various data quality issues with physician-rating websites.

**Objective:**

This study’s objective was to identify and categorize the data quality issues afflicting physician-rating websites by reviewing the literature on online patient-reported physician ratings and reviews.

**Methods:**

We performed a systematic literature search in ACM Digital Library, EBSCO, Springer, PubMed, and Google Scholar. The search was limited to quantitative, qualitative, and mixed-method papers published in the English language from 2001 to 2020.

**Results:**

A total of 423 articles were screened. From these, 49 papers describing 18 unique data quality issues afflicting physician-rating websites were included. Using a data quality framework, we classified these issues into the following four categories: intrinsic, contextual, representational, and accessible. Among the papers, 53% (26/49) reported intrinsic data quality errors, 61% (30/49) highlighted contextual data quality issues, 8% (4/49) discussed representational data quality issues, and 27% (13/49) emphasized accessibility data quality. More than half the papers discussed multiple categories of data quality issues.

**Conclusions:**

The results from this review demonstrate the presence of a range of data quality issues. While intrinsic and contextual factors have been well-researched, accessibility and representational issues warrant more attention from researchers, as well as practitioners. In particular, representational factors, such as the impact of inline advertisements and the positioning of positive reviews on the first few pages, are usually deliberate and result from the business model of physician-rating websites. The impact of these factors on data quality has not been addressed adequately and requires further investigation.

## Introduction

### Background

With the proliferation of mobile devices and instantaneous access to data, electronic word of mouth (e-WOM) has become a force to be reckoned with, affecting many aspects of our lives, including the things we buy, the shows we watch, and the places where we stay, directly or indirectly. Such dependence on e-WOM is especially true in the context of choosing a physician, as consumers historically have relied on word of mouth, including personal recommendations [1]. A simple check on Google Trends showed that the phrase “doctors near me” is now searched almost nine times more than it was 5 years ago; therefore, it is not surprising to see a rise in the number and scope of physician-rating websites (PRWs), which are peer-to-peer information-sharing platforms that patients use to share reviews and ratings of their health care providers. National survey data indicated that one in six Americans consult online ratings [2]. More than 30% of consumers compare physicians online before choosing a provider [3]. Emphasizing the impact of PRWs, one study [4] noted that 35% of patients selected physicians based on good ratings, while 37% avoided physicians with bad ratings. Another study found that patients consult PRWs as their first step in choosing providers [5] and that 80% of users trust online physician ratings as much as personal recommendations. Millennials, who account for more than half of PRW consumers, were found to exhibit a different behavior when they were unhappy with their health care services [6]. People aged 65 years or above were more likely to complain to doctors directly, while people aged 18 to 24 years were more likely to tell their friends. This emphasizes the evolution of PRWs into a platform for open and honest communication.

Although PRWs are less popular compared with rating websites in other domains, such as fast-moving consumer goods and e-commerce, they have high potential for growth. However, PRWs historically have lagged behind user expectations [7,8], and one of the contributing factors is end users’ lack of confidence in the data quality of PRWs. Furthermore, such data quality issues assume high importance, as poor data quality could affect consumers’ care choices adversely. Previous research has discussed individual issues in specific contexts [9-57]; however, there is a need for a study that presents a holistic perspective by investigating a comprehensive set of data quality issues found in PRWs. This study fills that literature gap by gleaning data quality issues from several previous studies and classifying them based on the data quality framework.

### Data Quality Framework

**Figure 1:**
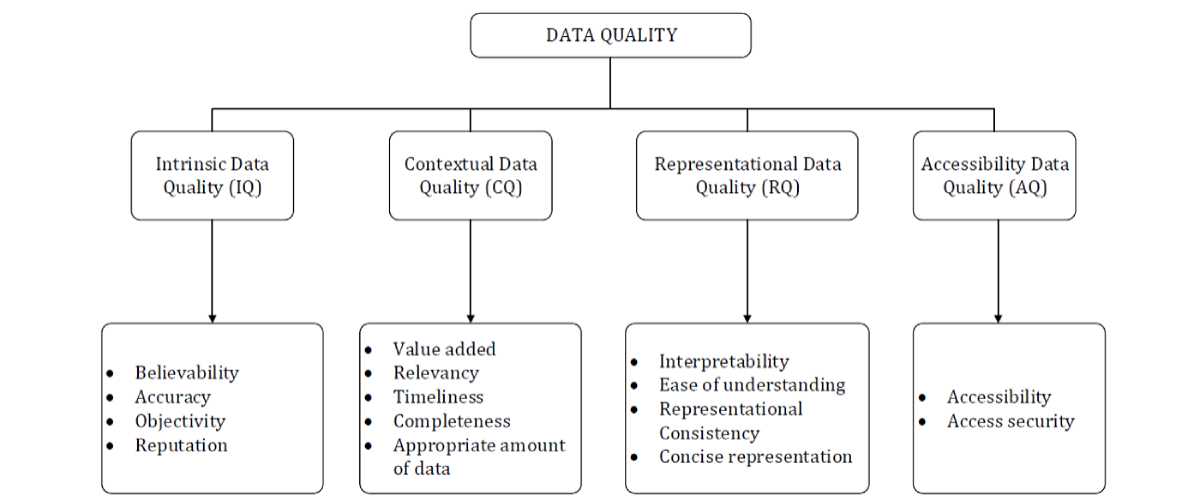
Data quality framework adapted from Wang and Strong [58].

We used a data quality framework developed by Wang and Strong [58] to classify data quality issues in PRWs. It was created by considering consumers’ perspectives on data quality, which accommodates a broader definition of data quality. Consequently, Wang and Strong defined *data quality* as “data that are fit for use by data consumers.” Furthermore, they empirically collected data quality attributes from consumers instead of determining these attributes theoretically or basing them on expert opinions to identify attributes that emerge from real consumers. They then used two-stage surveys and a two-phase sorting study to develop a hierarchical framework. They captured various data quality attributes, consolidated these attributes into dimensions, and distributed the dimensions across the following categories (Figure 1): intrinsic data quality (IQ), contextual data quality (CQ), representational data quality (RQ), and accessibility data quality (AQ).

IQ entails dimensions that are inherent to the nature of data, including accuracy, objectivity, reputation, and believability. While information system professionals typically have interpreted IQ to mean accuracy alone, consumers assess IQ broadly by considering other elements, such as reputation, objectivity, and believability of the source. CQ is a measure of data quality within the context of the task at hand. It includes dimensions, such as relevance, value addition, timeliness, and completeness, which are specific to a given situation. Together, RQ and AQ emphasize the role of systems that store the data. RQ underscores the importance of developing interfaces that concisely present data so that they are easy to understand and interpret. AQ focuses on making systems secure to ensure that data are safe and available only to relevant users. This framework also makes pragmatic sense, as consumers’ view of “fit for use data” would include data that are accurate, objective, believable, obtained from a reputable source, relevant to a specific task at hand, easy to understand, and accessible to them.

We use this framework because, unlike other frameworks [59], it accommodates a much broader definition of data quality. In addition, researchers have used it to evaluate the data quality of several customer-centric products, such as online bookstores and auction sites [60]. It has also been used to study critical factors affecting consumer-purchase behaviors in shopping contexts [61]. In recent years, as e-WOM has gained considerable prominence, some studies have used this framework to examine the impact of data quality on e-WOM [62]. Previous studies have used it to categorize data quality issues with electronic health records [63]. Furthermore, it has been used to study information quality issues on websites in which users, not experts, generate content [64]. Such an end-user point of view is especially relevant to our research, as most shortlisted studies discussed data quality issues in PRWs from patients’ perspective.

## Methods

### Overview

The study aimed to collect, analyze, and discuss data quality issues in PRWs based on the data quality framework of Wang and Strong. To accomplish this goal, we developed the following research questions:

- RQ1: What data quality issues exist in PRWs?
- RQ2: How are these data quality issues classified according to the Wang and Strong framework?
- RQ3: Which data quality issues have been addressed, and which ones warrant attention from researchers and practitioners?

**Figure 2:**
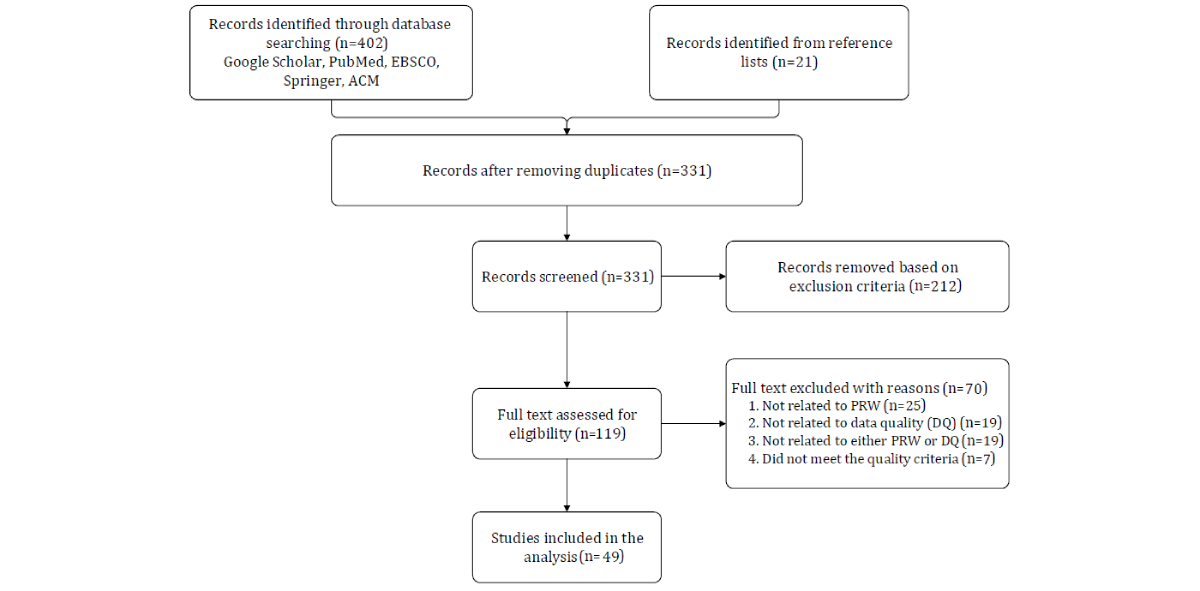
Literature search following the PRISMA guidelines. PRW: physician-rating website.

Following the Preferred Reporting Items for Systematic Reviews and Meta-Analyses (PRISMA) guidelines [65,66], we performed a systematic literature review (Figure 2).

### Search Strategy

We systematically searched for literature published in the past 20 years (between January 1, 2000, and January 1, 2020) using the following databases: ACM Digital Library, EBSCO, Springer, PubMed, and Google Scholar. The searches were performed using the following search terms: (“physician” OR “doctor” OR “provider”) AND (“review” OR “rating”) AND (“online” OR “internet”) AND (“data” OR “quality”). Initially, title, abstract, and index terms were used to screen for published journal articles, conference papers, proceedings, case studies, and book chapters. Two reviewers performed the screening independently. The reviewers met on a regular basis to discuss the inclusion of studies. A third reviewer was consulted when there was disagreement between the reviewers. Furthermore, the reviewers performed hierarchical searches by identifying literature sources through references cited in the shortlisted papers selected from the keyword searches to find additional relevant articles.

### Inclusion and Exclusion Criteria

*Physician quality* is an elusive concept to measure, as it means different things to different stakeholders. Health care professionals and policymakers have developed a plethora of clinical and process-quality measures to address the challenge of evaluating physician quality. Some well-known examples of such quality indicators include the risk-adjusted mortality rate [67], 30-day readmission rate [68], and percentage of patients receiving recommended preventive care. Although such clinical measures of quality are critical to improving the quality of care, they emphasize the process of care, not individual physicians’ quality. Furthermore, these measures are neither easy to access nor simple to understand. They also do not place high emphasis on patients’ perceptions of care quality. Owing to this lack of agreed-upon, meaningful, and readily available objective data on individual clinician performance, patients end up relying on other patients for recommendations.

PRWs fill this void by providing a platform on which patients can evaluate physicians based on their experiences; however, these ratings and reviews are individual patients’ subjective opinions and may not be indicative of physicians’ clinical quality. Thus, it is possible that a poorly rated physician has provided the correct or best treatment. Some studies argue that patients are not well-suited to evaluate physician quality because of the information asymmetry between patients and care providers [69]. In addition, several studies emphasize that ratings and reviews of patients are not correlated to clinical measures of physician quality [70,71]. Despite these shortcomings, PRWs have surged in popularity and have become instrumental in shaping prospective patients’ opinions. Individuals also use them to make crucial decisions, such as selecting a provider, because just as in any other consumer service business, customers’ perceptions impact revenue.

Therefore, in this paper, we focused on physician quality from patients’ perspectives, as captured by PRWs. Prior studies that examined data quality of patient-reported reviews and ratings were included in this literature review. Studies were excluded from this review if they (1) focused primarily on clinical quality measures that health care providers or public health agencies reported; (2) examined data quality issues that are not related to public PRWs (eg, papers that catered to paid websites, such as Castle Connolly, were excluded from this review); (3) were not available as full text in the final search; (4) were not written in English; and (5) were white papers, reports, abstracts only, letters, or commentaries.

### Data Extraction, Synthesis, and Evaluation

A Google document was created for data extraction. For each chosen study, the data collected included the title, author, year, country, abstract, study type, and number of participants. We assessed the selected studies’ quality based on the criteria listed in Table 1. The quality criteria were developed using guidelines specified by *the Cochrane Handbook for Systematic Reviews* [72] and the report “Guidelines for performing systematic literature reviews in software engineering” [73]. Two reviewers independently assessed every included study by assigning “Yes,” “No,” or “Cannot tell” scores to each criterion. Only studies that received a “Yes” on all criteria were included in this review. A senior researcher was consulted for a resolution if there was disagreement between the reviewers.

**Table 1:** Quality criteria for the included studies.

Identifier	Issue
C1	Does the article clearly show the purpose of the research?
C2	Does the article adequately provide the literature review, background, or context?
C3	Does the article present the related work with regard to the main contribution?
C4	Does the article have a clear description of the research methodology?
C5	Does the article include research results?
C6	Does the article present a conclusion related to the research objectives?
C7	Does the article recommend future research directions or improvements?

## Results

### Characteristics of Reviewed Studies

We included 49 papers published between 2009 and 2019. Among these, 28 articles were identified through the initial search and additional 21 articles meeting the study criteria were identified by reviewing the reference lists of those articles. The list of included papers is presented in Multimedia Appendix 1. We identified 18 unique data quality issues that afflict PRWs and classified these issues into four categories based on the data quality framework. In recent years, PRWs have captured the research community’s attention as evidenced by a surge in publications in the past 5 years. More than 71% (35/49) of the papers used quantitative methods to test their research hypotheses, 22% (11/49) adopted qualitative methods, and 6% (3/49) leveraged mixed methods. Healthgrades (n=12), RateMDs (n=8), Vitals (n=7), and Jameda (n=4) were the most targeted PRWs, while other sites, such as Zocdoc, Press Ganey, and Healthcare Reviews, were not examined as much. Three studies compared PRWs with business directory and review sites such as Yelp. In addition, several studies collected data from multiple PRWs to compare their results across different rating websites. Altogether, 26 articles focused on issues relating to IQ, while 30 discussed CQ concerns. Four articles emphasized RQ errors, and 13 were related to AQ challenges. Around half (26/49) the included studies focused on more than one type of data quality issue.

## Discussion

### Principal Findings

Consistent with this study’s goals, we discuss different data quality issues in a narrative format based on the four types of issues specified in the data quality framework. 

#### IQ Issues

As presented in Table 2, we discuss intrinsic data quality issues based on the following dimensions: accuracy, objectivity, believability, and reputation. The main hurdle affecting the accuracy of reviews and rating data was the glaring absence of negative ratings. A prior study highlighted the absence of negative ratings by empirically showing that physicians with low patient-perceived quality were less likely to be rated. Although a positive correlation between online ratings and physician quality was found, the association was the strongest for the medium segment. While the ratings were not sensitive for high-quality physicians, there were fewer ratings for physicians at the lower end of the quality distribution [9].

Another study showed the presence of ubiquitous high ratings for interventional radiologists, with mean ratings ranging from 4.3 to 4.5 on a five-point scale [10]. This lack of negative ratings was not only limited to interventional radiologists, but also spanned other medical specialties. Several studies corroborated this finding by noting that the average physician rating across all specialties was consistently very positive [11-16]. Sparse negative ratings could also be attributed to legal restrictions on entering negative feedback in some countries, such as Switzerland. However, this dearth of negative ratings adversely affects overall opinions about data quality greatly. 

**Table 2:** Intrinsic data quality issues.

Issues	Dimension	Citations
Ratings were either positive or extremely positive, with a notable absence of negative ratings.	Accuracy and objectivity	[9-16]
A significant number of ratings contained extreme values, typically in the form of a dichotomous distribution of the minimum and maximum values.	Objectivity	[9,17-19]
A significant number of reviews contained emotionally charged comments, implying a lack of objectivity in the reviews.	Objectivity	[20-22]
Online ratings were less sensitive to physician quality at the high end of quality distribution, implying the presence of the halo effect.	Objectivity	[9,23,24]
Some physician-rating websites did not ensure ratings’ accuracy by allowing anonymous ratings that were not entirely believable.	Believability	[18,20,25,26]
Some sites allowed premium-paying physicians to hide up to three negative comments.	Believability and reputation	[18,27,28]
Physicians were more likely to trust patient-experience surveys that health systems issued, whereas patients were more likely to trust ratings found on independent websites.	The data source’s reputation	[29]

Several research studies [9,17-19] questioned the objectivity of review and rating data by uncovering the high presence of extreme ratings, such as one- or five-star ratings. Extreme ratings do not represent a balanced view and are usually an impulsive response to an emotional trigger. Other studies [20-22] further corroborated these findings by revealing the presence of emotionally charged review comments. On one hand, physicians with low perceived quality were hardly rated; thus, a single negative review had a disproportionate impact on the overall rating. On the other hand, physicians with several reviews incurred no relevant impact from a negative rating on overall numbers owing to relatively few negative ratings. Two studies also highlighted the presence of the halo effect [23,24]. One found that higher ratings were associated with marketing strategies that the physicians employed. It also discovered that physicians’ online presence greatly impacted their ratings. This phenomenon demonstrates the susceptibility of reviews and ratings to external factors, such as marketing and promotion, casting serious doubt on the credibility of the data.

Other studies [18,20,25] examined data quality challenges that emerge when users can rate physicians anonymously. Anonymity exposes information to manipulation from sources such as competition, slanderers, and biased friends. The effect of anonymous ratings cascades into a relevant issue when the number of genuine negative ratings is small, as is the case with PRWs. An excellent example of such abuse of PRWs is found in how antiabortion groups deliberately target physicians working in abortion clinics with libelous comments and negative ratings under the veneer of anonymity [26].

Several business models of PRWs also skew the believability of ratings and review information by providing physicians with premium subscriptions having an option to hide up to three negative comments [27,28]. The hidden negative reviews may mislead consumers who are usually unaware of the business models of PRWs. Several researchers also questioned the ethics of hiding up to three negative ratings when, on average, there were less than three negative physician ratings [18].

#### RQ Issues

As presented in Table 3, RQ emphasizes clear representation and includes dimensions such as interpretability, ease of understanding, representational consistency, and conciseness. Of all data quality issues, the ones related to RQ are the most insidious, as even an accurate data set can lead to misleading conclusions if representation is not appropriate. One study observed how users can be influenced by the mere repositioning of positive reviews to the first few pages, as most users read initial reviews more than subsequent ones. The same study examined the negative impact of placing poor ratings at the top of the profile page [25]. Furthermore, researchers have argued that the five-point scale that PRWs use is an imperfect proxy for physician quality [30,31] as the difference between a rating of 4.8 and 4.9 might be too small to be meaningful from an end user’s perspective. These RQ issues seem to be more deliberate than the other data issues and result from the business or revenue model of PRWs. Thus, they may be more challenging to overcome.

**Table 3:** Representational data quality issues.

Issues	Dimension	Citations
The five-point scale used for measuring physician quality did not have the finer granularity needed to highlight the minor differences in physician quality.	Interpretability	[30,31]
The positioning of positive reviews and rating data on the first few pages greatly impacted patient perceptions.	Representational consistency	[25]
Every physician-rating website used different underlying scales to measure the effectiveness of the physicians. Therefore, interpreting results across different physicians can be difficult.	Interpretability	[32]

#### CQ Issues

As presented in Table 4, CQ examines issues in the context of the task at hand. For this paper, the task is assumed to be patient decision making in terms of choosing a provider by analyzing ratings and reviews. Several data quality issues plagued this dimension. The most fundamental concern stemmed from the need for an appropriate amount of data to make meaningful decisions. One critical issue in this segment was the nonexistence of ratings and reviews for most of the physicians. Several papers [22,23,33-37] found that more than half of the physicians had no ratings or reviews. They also argued that no meaningful decisions could be made with the unavailability of data for such a considerable volume of physicians. Another related threat to CQ was the low volume of reviews and ratings. One study [23] noted that 57% of the doctors received only one to three ratings. Such low volumes cast doubts on the ratings’ credibility, especially when sites allow anonymous ratings. Previous research showed that users did not trust the rating and review data, and sought information from alternative sources until a minimum number of ratings was available. Thus, it should come as no surprise that multiple prior studies on e-WOM in other domains, such as e-commerce, found that high volumes lend more credence to rating and review data. Furthermore, data analysis showed that early negative reviews beget more negative reviews [25]. At the same time, doctors with great initial reviews might continue to benefit from these reviews, even if their clinical quality has declined over the years.

A nuanced challenge to CQ emerged from the underlying factors used to compute ratings and reviews. One study [51] compared the factors between two sites, one from the United States and another from Germany. They found that German PRWs focused on parameters that measure physician characteristics, while American sites focused on the entire clinical process, including registration, clinical pathways, and staff behaviors. Typically, most PRWs include wait times, staff behaviors, follow-ups, and ease of making appointments, some of which are not under physicians’ direct control. In addition, these factors may not be truly representative of physician quality. Some studies suggested taking reviews and ratings with a “grain of salt,” as the ratings reflected patients’ perceptions and did not objectively measure physician quality.

Furthermore, patients might not be able to assess a wide range of physician attributes owing to information asymmetry between physicians and patients. Some studies have discussed the possibilities of bringing both patient perspectives and clinical quality measures together to enhance CQ [38]. The ease of decision-making from a user’s perspective defines the essence of CQ. Such a user perspective was affected adversely when a low degree of correlation existed among different physician review websites [44,48-51], as users may not know which websites to trust.

**Table 4:** Contextual data quality issues.

Issues	Dimension	Citations
There was a low volume of reviews and ratings, with more than half the physicians having less than one to three ratings.	Appropriate amount of data	[22,23,33-37]
Physician-rating websites captured patient perceptions of physician quality; they did not capture and present objective measures of quality, such as Physician Quality Reporting System (PQRS) ratings for physicians or risk-adjusted mortality rate.	Objectivity completeness	[29,31,38,39]
Positive ratings were based on factors, such as ease of getting an appointment, short wait times, and staff behaviors, that did not directly represent physician characteristics.	Relevance	[21-23,29,41-46]
Higher ratings were associated with marketing strategies that physicians employed, such as significant online presence and promotion of satisfied patients’ reviews.	Objectivity relevance	[40,43,47]
There was a low degree of correlation among online websites on surgeon ratings.	Value addition	[44,48-51]

#### AQ Issues

As presented in Table 5, AQ focuses on the dimensions ease of access and security of access. While PRWs are afflicted by only a limited set of accessibility challenges, some issues warrant further discussion. First, while the internet may be accessible universally, we must consider socioeconomic and psychographic barriers to the accessibility of PRWs [53]. Typically, tech-savvy people with reasonable income and education use PRWs. Several studies noted that PRWs did not represent the opinions of elderly people, who comprise the largest consumer segment for health care services. Second, the number of ratings and reviews that physicians received depended on their specialty. One study noted that physicians in specialties that warranted high interaction with patients, such as obstetrics and gynecology, were more likely to be rated, while other specialties, such as pathology, were less likely to be rated [54].

Several studies found that the maturity level of PRWs was not uniform across countries. While PRWs have been adopted widely in the United States, the United Kingdom, and Canada, they were at an early stage of adoption in other countries, such as Australia [34], Switzerland [55], Lithuania [56], and Germany [57]. Data quality issues with PRWs in these countries were more pronounced when compared with other nations. Furthermore, accessibility issues emerged owing to legal and regulatory challenges. For instance, Switzerland provides physicians with a legal option to have negative reviews deleted.

**Table 5:** Accessibility data quality issues.

Issues	Dimension	Citations
The frequency and volume of ratings varied greatly based on physician specialty; therefore, some specialists’ ratings might not have been easily accessible.	Ease of accessibility	[21,52]
Even though the internet was widely accessible, financial and social access barriers had to be considered. Such barriers include income, culture, gender, and age. The effective use of physician-rating websites remained primarily dependent on users’ cognitive and intellectual capabilities.	Accessibility	[37,51,53,54]
The maturity of physician-rating websites was inconsistent across countries. Physician-rating websites were in the early stage of adoption, with very few ratings in many countries, such as Lithuania and Australia.	Appropriate amount of data completeness	[34,49,53-57]

### Opportunities for Future Research

In this review, the key observation was the lack of emphasis on RQ and AQ issues in prior research. Most studies highlighted IQ and CQ issues, which are foundational to achieving other types of data quality. Although the number of papers published on RQ [30-32] and AQ [55-57] issues has increased in recent years, more research is warranted on these issues.

Specifically, the misleading impact of inline advertisements and the effect from framing reviews differently warrant further investigation. Furthermore, while the application of data analytic methods has surged considerably, there is a conspicuous absence of studies that have examined the impact of different kinds of visualizations of rating and review data on patient decision-making. Similarly, machine learning and predictive analytic methods could well be used to forecast future physician ratings. Such studies could educate physicians further on strategies to improve their future ratings. Another observation is the need to examine the impact of physician reviews and ratings on providers’ revenue or insurance payments. Understanding this impact can help explain physicians’ behaviors and motivations.

Relatively few studies have discussed the use of data analytics, machine learning, and other statistical methods to identify and distinguish fake reviews from genuine ones. The development of such mechanisms can substantially enhance the quality of review and rating data. Further, data privacy laws, such as General Data Protection Regulation (GDPR), can potentially impact the quality of review and rating data adversely. More research is warranted to examine the impact of these regulations on accessibility data quality.

### Limitations

This systematic review has several limitations. The first is associated with the methodology used to select studies. Relatively few (n=49) studies were included, based on the selection criteria that the authors set. Five databases were searched to select studies. Other databases potentially could have yielded additional studies. Furthermore, while the authors performed due diligence in execution, the search was limited to the set of selected keywords. It is possible that some relevant studies were not identified owing to keyword mismatches or title differences and, therefore, were not included in the review.

Second, we only included articles written in the English language, and business models can vary greatly among geographical locations, depending on existing practices, cultures, and regulations. We tried to find literature from diverse geographical areas, but few studies were accessible to us owing to limitations caused by language barriers and the extent of research in other geographical areas. Furthermore, we did not include non–peer-reviewed sources, such as white papers and dissertations. It is possible that relevant information from such sources could have influenced our findings.

### Conclusion

This paper contributes to a better understanding of data quality issues in PRWs by highlighting several vital challenges that these issues pose. The paper acknowledges the tremendous potential that PRWs have in transforming health care by being the voice of consumers and increasing the transparency of health care processes. However, this study showed that data quality challenges present relevant hurdles to the realization of these benefits. The impact of these data quality issues will only surge as millennials base their decisions on PRW data.

Historically, IQ and CQ factors have been principal sources of data quality issues, and many researchers have studied them extensively. However, RQ and AQ factors warrant more research. In particular, RQ factors, such as the impact of inline advertisements and the positioning of positive reviews on the first few pages, are usually deliberate and result from the business or revenue model of PRWs. In addition, data privacy regulations, such as GDPR in the European Union and California Consumer Privacy Act (CCPA) in California, may greatly impact PRWs. More research is needed to understand their implications. Furthermore, the effect of cultural factors (eg, in some cultures, speaking negatively about authority figures is viewed as inappropriate), though relevant, was not considered, as it is under-addressed in the literature. Future innovations and research are needed to address these emerging data quality issues. We hope that this study’s results inspire professionals and researchers to develop PRWs that are more robust and do not have many data quality issues.

## Appendix

Multimedia Appendix 1 List of all included studies.

## Abbreviations

AQ: accessibility data quality
CQ: contextual data quality
e-WOM: electronic word of mouth
GDPR: General Data Protection Regulation
IQ: intrinsic data quality
PRW: physician-rating website
RQ: representational data quality
